# Assessment of G10 Intraoperative Scoring System for Conversion in Patients Undergoing Laparoscopic Cholecystectomy: A Cross-Sectional Study From Nepal

**DOI:** 10.7759/cureus.55392

**Published:** 2024-03-02

**Authors:** Suman Baral, Neeraj Thapa, Shrinit Babel, Shasi Poudel, Raj K Chhetri

**Affiliations:** 1 Surgery, Lumbini Medical College and Teaching Hospital, Tansen, NPL; 2 Morsani College of Medicine, University of South Florida, Tampa, USA; 3 Emergency Medicine, Fewa City Hospital, Pokhara, NPL

**Keywords:** cholecystitis, g10 protocol, laparoscopic conversion, conversion surgery, lap cholecystectomy

## Abstract

Introduction: Various preoperative risk factors for conversion in laparoscopic cholecystectomy (LC) have been well studied. However, the assessment of intraoperative factors for conversion in patients with cholecystitis is unclear. The G10 scoring system, which incorporates 10 parameters, has tried to fill this void by developing a scoring system for the most commonly encountered surgical illnesses. So, we aimed to assess the utility of the G10 scoring system among patients presenting for LC for symptomatic cholelithiasis (both acute and chronic cholecystitis) in the clinical setting of a low- and middle-income country.

Methods: All the patients undergoing LC were assigned a G10 value. Gallbladder surgery was considered easy if the G10 score was <2, moderate (2 ≦ 4), difficult (5 ≦ 7), and extreme (8 ≦ 10). All 10 risk factors were analyzed into a binary logistics model, and statistically significant risk factors were assessed.

Results: Among 177 patients, there were 36 males and 141 females. The median age of the patient was 42 years (range 11-79). There were 70 easy, 89 moderate, and 18 difficult cases. The overall mean G10 score was 2.32±1.5, which significantly increased as the severity progressed, with a mean value of 5.5±0.51 for difficult cases (P=0.0001). The mean G10 score for surgeries completed laparoscopically was 2.1±1.4, while it was 3.71±1.4 for open conversions [P=0.0001, AUC=0.79, CI=0.70-0.87]. There were 18 patients with G10 ≥5 with a conversion rate of 27.7%, while the overall conversion rate was 13.6%. Multivariate analysis showed free bile or pus outside the gallbladder [P=0.02, OR=5.1, CI=1.2-21.1] and fistula [P=0.01, OR=15.8, CI=1.9-129.8] as significant risk factors for conversion.

Conclusion: Intraoperative risk factors for the prediction of conversion included the presence of free bile or pus outside the gallbladder and cholecystoenteric fistula. Based on the F1 score analysis, complemented with the Youden Index, the optimal cutoff value for conversion, based on the G10 score, lies around 4. Broader application and validation of the G10 scoring system are mandated to assess the utilization of this novel intraoperative scoring system.

## Introduction

Laparoscopic cholecystectomy (LC), one of the most common surgical procedures done worldwide in elective and emergency settings, has been a gold standard for treating symptomatic gallstone diseases [1]. Though this procedure has been regarded as one of the easiest for amateurs of laparoscopy, its complications can be troublesome to the patient and treating surgeons. A healthy gallbladder, also termed “robin’s egg blue,” is not always encountered during surgery: repeated attacks of biliary colic and cholecystitis might have rendered the gallbladder to change morphologically, where inflammatory changes predispose difficulties, leading to a contracted and fibrosed gallbladder, termed as chronic cholecystitis changes. Similarly, anatomical variation and anomalies may create confusion, even for experienced surgeons, and acute presentations may make surgeries difficult, elevating the chances of conversion. Based on the surgeon’s expertise, different bailout procedures could be enacted. However, an open conversion could be anticipated in many instances, especially in low-resource settings, followed by even a subtotal cholecystectomy. Various preoperative risk factors for conversion have been mentioned in the literature. Regardless, most of them do not consider intraoperative anatomical differences [2-5]. Other scoring systems like Tokyo Guidelines 2013, the AAST severity scoring system for cholecystitis, and the Parkland Grading system have been established. However, their robust use is still not implemented owing to a lack of validation, simplicity, and relevance. However, updates are being released to enhance practicality [6,7]. A 10-point cholecystitis severity score, termed the G10 scoring system, was devised by Sugrue et al. in 2015 and revised in 2019. It is based on the intraoperative appearance of the gallbladder, distension/contraction, accessibility, and associated sepsis and complications encountered while performing LC [8]. Therefore, this cross-sectional observational study aimed to evaluate the effectiveness of this intraoperative scoring system in predicting surgical outcomes and the risk factors associated with conversion related to G10 scoring among patients operated in a rural part of the country. We believe introducing and assessing surgical patients intraoperatively with this scoring system renders the patient an early conversion and reduced operative time with diminished chances of postoperative injury. 

## Materials and methods

A cross-sectional observational study was conducted in the Department of Surgery, Lumbini Medical College Teaching Hospital, after authorization from the Institutional Review Committee of the institute. The ethical approval number was IRC-LMC 08-G/019. The study lasted eight months, from 20th August 2019 to 20th April 2020, and written informed consent was obtained from every participant. A special proforma was designed that included all the variables, such as demographics, laboratory parameters, patient age, gender, type of surgery, elective or emergency LC, open conversions and sub-total cholecystectomies when undertaken, and duration of surgery. All patients with symptomatic gallstone disease, including acute and chronic calculus cholecystitis, were included in the study. Patients who were excluded included those who were unfit for general anesthesia, had acute calculus cholecystitis managed conservatively, and had a history of chronic liver disease, choledocholithiasis, and features of obstructive jaundice. A modified 10-point intraoperative gallbladder scoring system (G10), devised by Sugrue et al. in 2019, was used. The variable assessing the time to identify the cystic artery and duct, if exceeding 90 minutes, was removed. In its place, the presence of a cholecystoenteric fistula was added as a parameter specifically targeting sepsis and complications. G10 focuses on four key components: the intraoperative appearance of the gallbladder, distension or contraction of the gallbladder, ease of access, and the presence of sepsis in the peritoneal cavity, either biliary peritonitis or purulent fluid and/or a cholecystoenteric fistula. All these components, as part of the G10 scoring system, were assessed and documented by the accompanying surgeon in the operation theater. Notably, the main operating surgeon remained blinded to the G10 score calculated at that instant, as well as to the primary objective of the study -- the decision to convert to open surgery. Meanwhile, the accompanying surgeon, who was not scrubbed in, recorded the G10 score in the proforma. The decision to convert was solely made by the operating surgeon based on their assessment of the intraoperative conditions. This separation of responsibilities aimed to address information bias and the Hawthorne effect, ensuring an unbiased evaluation of the G10 components. Three faculty surgeons, each with over three years of experience, were involved in the surgeries. Descriptive data were presented as mean and standard deviation. Student’s t-test was applied for continuous quantitative variables, while chi-square/Fisher's exact test was applied for categorical variables. Statistical analysis was performed with Statistical Package for Social Sciences (SPSS^TM^) software version 20 (Armonk, NY: IBM Corp). Univariate analysis was applied for 10-point risk factors of the G10 scoring system considering significance; if the P-value was less than 0.05, that determined conversion while performing LC. Significant risk factors in univariate analysis were further processed with binary logistics regression in a multivariate analysis model, and their statistical significance was assessed. The area under the receiver operating characteristic (ROC) curve with a 95% confidence interval was used to find the diagnostic and predictive value of the intraoperative score for predicting the outcome that determined the accuracy of the G10 cholecystitis severity score. Gallbladder surgery was considered easy if the G10 score was <2, moderate (2 ≤ 4), difficult (5 ≤ 7), and extreme (8≤ 10). This stratification of the difficulty level was done as per the G10 score by Sugrue et al. [9]. Finally, to explore the optimal cutoff value following the G10 score, both the F1 score and the Youden Index were analyzed. The F1 score measures the harmonic mean of precision and recall for different G10 score thresholds; a balance between precision and recall suggested a better threshold. Likewise, the Youden Index was also used to illustrate the maximum potential effectiveness at set thresholds. 

## Results

Table 1 shows the patient demographics along with intraoperative characteristics.

**Table 1:** Patient Demographics and Intraoperative Characteristics *Intraoperative Bleeding: *Intraoperative bleeding included arterial sprouting or sinus bleeding that required intervention such as cauterization, clipping of the bleeder, compressing of the bleeding site with gauze, or involvement of the gallbladder itself. *Postoperative Retained Stone: *For one patient, the stone was in the cystic stump, and for two others, it was in the gallbladder.

Variables	Overall	Easy N=70	Moderate N=89	Difficult N=18	P-Value
Male:Female (M/F)	36:141	11:59	21:68	4:14	
Mean age (years)±SD	47.55±17.6	47.6±17.8	47.9±18.1	45.3±14.5	0.84
Mean G10 score±SD	2.32±1.5	0.94±0.23	2.75±0.84	5.5±0.51	0.0001
Duration of surgery (min)±SD	58.9±26.1	50.93±17.38	61.24±28.4	78.33±31.5	0.0001
Open conversion	24	1	18	5	
Sub-total cholecystectomy	6	0	2	4	
Intraoperative bile spillage	17	3	10	4	
Intraoperative bleeding	23	0	13	10	
Postoperative retained stone	3	0	0	3	
Postoperative pancreatitis	2	0	0	2	

A total of 190 patients presented to the hospital with features of symptomatic cholelithiasis. However, 13 of them were excluded. Among 177 patients who underwent cholecystectomy, 36 were males and 141 were females. The median age of the patient was 42 years (range: 11-79). The mean G10 score significantly increased as the severity increased, with a maximum mean value attained of 5.5±0.51 for severe cases (P=0.0001). The median G10 score was 2. The mean duration of surgery showed a significant increase as the difficulty level increased. Six patients underwent open subtotal cholecystectomy through the reconstituting approach. Intraoperative bleeding occurred in 23 patients; however, it was easily controlled. There was no evidence of bile duct injury. Every open case had drains, and no postoperative bile leaks/bile were detected in the drain. Three patients had postoperative retained stones, while two patients developed acute pancreatitis, which was managed conservatively. There was no mortality.

Table 2 shows the conversion rates while the G10 score increases. Most of the patients had G10 scores of 1 to 4. The maximum score value was 6, which included nine patients, with four conversions and a conversion rate of 44.4%. Similarly, there were 18 patients with G10 ≥5, which showed five conversions with a conversion rate of 27.7%. However, the overall conversion rate among 177 patients was 13.6%. 

**Table 2:** Computed G10 Scores and Conversion Rates

G10 score	Frequency (N)	Conversion to open cholecystectomy (%)	
		No	Yes
0	4	100	0
1	66	98.5	1.5
2	45	88.9	11.1
3	21	85.7	14.3
4	23	56.5	43.5
5	9	88.9	11.1
6	9	55.6	44.4

**Figure 1:**
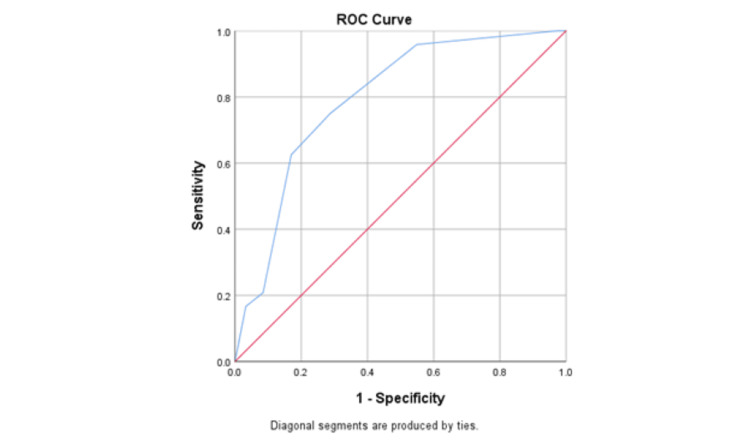
Receiver Operating Characteristic (ROC) and Area Under the Curve (AUC) for Prediction of Intraoperative Difficulty and Conversion Based Upon Intraoperative G10 Scores

Table 3 depicts the relationship between various risk factors as described by the G10 scoring system in relation to intraoperative outcome (conversion vs. non-conversion) while performing cholecystectomy. All factors, namely the appearance of the gallbladder, distension/contraction of the gallbladder, accessibility for surgery, and sepsis and related complications, were studied and tabulated. Univariate analysis showed a completely buried gallbladder (P=0.02, OR=2.9, CI=1.15-7.4), inability to grasp without decompression (P=0.002, OR=4.5, CI=1.7-11.7), impacted stone >1 cm (P=0.03, OR=3.4, CI=1.06-10.8), free bile or pus outside the gallbladder (P=0.001, OR=7.4, CI=2.4-23.1), and fistula (P=0.012, OR=10.7, CI=1.7-68.3) as a significant risk factor for conversion while performing LC. Multivariate analysis showed free bile or pus outside the gallbladder (P=0.02, OR=5.1, CI=1.2-21.1) and fistula (P=0.01, OR=15.8, CI=1.9-129.8) as significant risk factors for conversion at our clinical setting. ROC curve analysis showed a significant association between G10 value and conversion. Overall, the mean G10 score for surgeries completed laparoscopically was 2.1±1.4 while it was 3.71±1.4 for groups who underwent conversion to open cholecystectomy (P-value: 0.0001), which was statistically significant. At a 95% CI, the AUC was 0.79 with a P-value of 0.0001 (Figure 1). 

**Table 3:** Univariate and Multivariate Analyses of Various Risk Factors Related to G10 Scoring System Regarding the fistulas, five patients had cholecystoduodenal fistulas. Two patients underwent laparoscopic interrupted closure of the duodenal fistula, while three were converted to open surgery, undergoing single-staged interrupted suture closure with omental patching. No postoperative bile leaks were observed. GB, gallbladder.

Risk factors	Level	Intraoperative outcome		Univariate	Multivariate
		No conversion	Conversion		
Gallbladder appearance	Adhesion <50% of GB	79	8	P=0.1, OR=0.46 (0.18-1.15)	
	Adhesion >50% of GB	19	6	P=0.1, OR=2.3 (0.83-6.6)	
	Completely buried GB	26	9	P=0.02, OR=2.9 (1.15-7.4)	
Distension/contraction	Distended/shrivelled GB	81	15	P=0.38, OR=1.4 (0.61-3.6)	
	Inability to grasp without decompression	18	9	P=0.002, OR=4.5 (1.7-11.7)	
	Stone >1 cm impacted	11	5	P=0.03, OR=3.4 (1.06-10.8)	
Access	BMI >30	2	2	P=0.06, OR=6.8 (0.9-51.2)	
	Adhesions from previous surgery	4	1	P=0.67, OR=1.6 (0.17-15.1)	
Sepsis and complications	Free bile or pus outside GB	8	7	P=0.001, OR=7.4 (2.4-23.1)	P=0.02, OR=5.1 (1.2-21.1)
	Fistula	2	3	P=0.012, OR=10.7 (1.7-68.3)	P=0.01, OR=15.8 (1.9-129.8)

**Figure 2:**
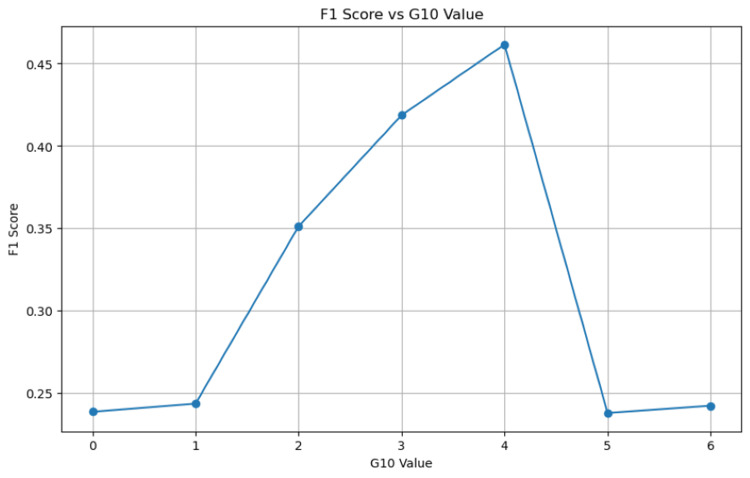
F1 Score vs G10 Value

Figure 2 plots the F1 scores as a function of various G10 thresholds. The Youden Indices for every threshold, to complement the F1 analysis, are tabulated in Table 4. 

**Table 4:** Youden Index Calculated for Various G10 Thresholds

Threshold G10 value	Youden index
7	0.000000
6	0.133987
5	0.123366
4	0.455065
3	0.462418
2	0.409314
1	0.026144
0	0.000000

## Discussion

Conversion during LC should be viewed as a result of the surgeon's sound clinical judgment rather than a surgical failure, considering the presence of various intraoperative and patient factors [10]. Studies are increasingly examining the factors responsible for the conversion, albeit most analyze preoperative factors and predictors of conversion. Indeed, the preoperative prediction makes the operating surgeon cautious, with adequate preparation for patient parties regarding possible outcomes, heeding advice from colleagues in case of difficulty or arriving at a surgical decision. That being said, it does not appear reasonable to undergo open surgery with a right subcoastal scar without pursuing laparoscopy, considering the presence of preoperative risk factors in a specific cohort of patients. Further, surgeon factors may also be responsible for the conversion, including the experience years or the number of surgeries performed, experience with more complex cases, level of perseverance regarding conversion, or intraoperative events (e.g., suspected bile duct injury or bleeding) [11,12].

Vivek et al., in their prospective study of 323 patients, concluded that clinical parameters like male gender, previous history of cholecystitis and upper abdominal surgery, and radiologic parameters (including multiple stones along with peripancreatic fluid collection) are risk factors for conversion. Also, intraoperative parameters like cirrhotic liver contracted/distended gallbladder, ductal anomalies, and associated adhesions were significant findings [13]. Likewise, Nachnani et al. identified male gender, previous abdominal surgery, BMI >30, previous acute cholecystitis/acute pancreatitis along with a gallbladder wall thickness of more than 3 mm with a conversion rate of 11.4% in the cohort of 105 patients. This parallels our context, as India and Nepal share similar sociocultural, nutritional, geographic, and population health features and potentially similar problems impacting surgical outcomes [14]. A study from Delhi, which included 73 patients who underwent LC, saw a conversion rate of 23.3% with a positive predictive value of gallbladder wall thickness >4 mm, impacted stone at Hartmann's pouch, and contracted gallbladder to predict conversion to be 70%, 63.6%, and 45.4%, respectively [15].

The Parkland grading system for cholecystitis stratifies the severity of gallbladder disease by classifying the cohorts into a five-tiered grading system based solely on anatomical and inflammatory factors. These factors vary among patients, and these stratifications were done intraoperatively. A total of five grades were formulated, hypothesizing that changes in gallbladder anatomy and inflammation would relate to the perioperative outcomes. Also, this grade has recently been validated by the proposer authors in 317 gallbladders with statistically significant factors, such as acute cholecystitis, surgical difficulty, the incidence of a partial and open cholecystectomy, preoperative WBC, length of surgery and bile leak rates, which showed increment with increasing grades [16]. However, further validation studies are needed to facilitate the significance of this study. Updated Tokyo Guidelines 2018 (TG 18) classified cholecystitis into three grades: mild (Grade 1), moderate (Grade 2), and severe (Grade 3), and ascertained treatment modalities according to the grades. For Grade I, TG18 recommended early LC if the patients met the criteria of Charlson comorbidity index (CCI) ≤5 and American Society of Anesthesiologists physical status classification (ASA-PS) ≤2. For Grade II, if patients met the criteria of CCI ≤5 and ASA-PS ≤2, TG18 recommended early LC performed by experienced surgeons; if not, LC was indicated after medical treatment and/or gallbladder drainage. Similarly, Grade III patients needed to fulfill strict criteria, including a favorable organ failure profile and negative predictive factors. These criteria involved fulfilling the requirements of CCI ≤3 and ASA-PS ≤2, and receiving treatment at an advanced center with experienced surgeons. If the patient was not considered suitable for early surgery, TG18 recommended early/urgent biliary drainage followed by delayed LC once the patient's overall condition improved [17]. However, TG did not consider intraoperative factors and operative findings when reporting outcomes. Similarly, the latest Tokyo consensus emphasized that conversion should never be considered a complication, as it is in the best interest of patient safety [18]. 

Our present study identified other risk factors, such as a completely buried gallbladder during the initial view after inserting the telescope, difficulty in grasping the gallbladder without decompression of the bile, impacted the stone size of more than 1 cm, free bile or pus outside the gallbladder, and the presence of a cholecystoenteric fistula. While tabulating the individual potential of predicting the conversion (P<0.05), however, the binary logistics regression (when applied for statistically significant variables) and multivariate analysis nullified the above factors, except for cholecystoenteric fistula and free bile or pus outside gallbladder as clinically significant risk factors. Our experience suggests that cholecystoenteric fistula is one of the most difficult clinical conditions to be diagnosed preoperatively and managed once they get diagnosed intraoperatively. We encountered five similar cases where fistulas with the duodenum were created. Two of these cases were successfully managed laparoscopically with subtotal cholecystectomy, while the remaining three required conversion and suture closure in layers. No bile leaks were demonstrated postoperatively.

The AUC for the G10 score was 0.79 in this study, which resembles the AUC of 0.77 demonstrated by Sugrue et al. [9]. This similarity supports the external validity of applying their scoring system to low- and middle-income countries (LMICs), where the disease course might be more advanced compared to high-income countries. Factors contributing to this advanced disease progression in LMICs include financial constraints, lack of knowledge about the condition, pursuing home-based treatments, and topographical challenges, such as limited travel access [19].

While exploring the optimal cutoff value following the G10 metric, the F1 score analysis suggested that a G10 score of 4 best balances precision and recall and, therefore, best predicts conversion. The Youden Index, following a distinct analytical approach, also corroborated this aspect. The agreement with the F1 score analysis and Youden Index suggests that a G10 score of 4 is a potential threshold in predicting the need for conversion for this dataset. However, further studies are needed for validation, as the robustness of this cutoff value may vary by context.

This study has some limitations. As most of the guidelines are proposed and formulated in developed nations, taking population differences into account, their applicability to LMICs could be limited, where the clinical scenario might be significantly different. However, ongoing efforts in external validations and assessments of such clinical guidelines have yielded significant results, too. Adequate data collection tools, web-based registration mechanisms, development of research culture, and collaborative, active participation from LMICs are needed to improve the external validity of such surgical guidelines globally. Observational bias may be present due to inter-observer error when three of the surgeons were involved, although studies have shown poor reproducibility for inter-observer variability [9]. This is a single-center study involving smaller cohorts, which might not fully capture the clinical aspects of the entire national population. Nevertheless, considering the rural location of the study site, these shortcomings were addressed in the best possible way. All in all, this calls for more validation studies to replicate the efficacy of the G10 scoring system and measure its applicability in a global context.

## Conclusions

To conclude, the G10 scoring system proved to be an effective measure in demonstrating intraoperative risk factors for conversion in LC. Intraoperative factors, like free bile or pus outside the gallbladder and the presence of cholecystoenteric fistula, statistically predicted conversion in a low-resource setting. G10 score ≥5 showed conversion in 27.7% of patients. 
